# Author Correction: Dysregulation of MMP2-dependent TGF-ß2 activation impairs fibrous cap formation in type 2 diabetes-associated atherosclerosis

**DOI:** 10.1038/s41467-025-56969-6

**Published:** 2025-02-12

**Authors:** Pratibha Singh, Jiangming Sun, Michele Cavalera, Dania Al-Sharify, Frank Matthes, Mohammad Barghouth, Christoffer Tengryd, Pontus Dunér, Ana Persson, Lena Sundius, Mihaela Nitulescu, Eva Bengtsson, Sara Rattik, Daniel Engelbertsen, Marju Orho-Melander, Jan Nilsson, Claudia Monaco, Isabel Goncalves, Andreas Edsfeldt

**Affiliations:** 1 Cardiovascular Research–Translational Studies, Lund University, Malmö, Sweden; 2 Department of Clinical Sciences Malmö, Lund University, Malmö, Sweden; 3 Department of Biomedical Science, Malmö University, Malmö, Sweden; 4 Biofilms–Research Center for Biointerfaces, Malmö University, Malmö, Sweden; 5 Kennedy Institute of Rheumatology, Nuffield Department of Orthopaedics, Rheumatology and Musculoskeletal Sciences, University of Oxford, Oxford, UK; 6 Department of Cardiology, University Hospital of Skåne, Lund/Malmö, Sweden; 7 Wallenberg Centre for Molecular Medicine, Lund University, Lund, Sweden

**Keywords:** Atherosclerosis, Translational research, Carotid artery disease

Correction to: *Nature Communications* 10.1038/s41467-024-50753-8, published online 9 December 2024

In the version of the article initially published, in Supplementary Fig. 13a, the TGF β3, SMAD3 image was a duplicate of the TGF β1, SMAD3 image. A corrected version of the Supplementary information is now available with the online version of the article.

## Supplementary information

Updated Supplementary Information

