# Supplementary material for: Dysregulation of MMP2-dependent TGF-ß2 activation impairs fibrous cap formation in type 2 diabetes-associated atherosclerosis
Source: Nat Commun. 2024 Dec 9;15:10464. doi: 10.1038/s41467-024-50753-8 (PMC11628557; doi:10.1038/s41467-024-50753-8)
Supplement: Supplementary file 3 — Reporting Summary [file 41467_2024_50753_MOESM3_ESM.pdf]

Corresponding author(s): Andreas Edsfeldt

Last updated by author(s): Jun 19, 2024

Reporting Summary

Nature Portfolio wishes to improve the reproducibility of the work that we publish. This form provides structure for consistency and transparency in reporting. For further information on Nature Portfolio policies, see our Editorial Policies and the Editorial Policy Checklist.

Statistics

For all statistical analyses, confirm that the following items are present in the figure legend, table legend, main text, or Methods section.

n/a	Confirmed	
☐	☒	The exact sample size (*n*) for each experimental group/condition, given as a discrete number and unit of measurement
☐	☒	A statement on whether measurements were taken from distinct samples or whether the same sample was measured repeatedly
☐	☒	The statistical test(s) used AND whether they are one- or two-sided<br>*Only common tests should be described solely by name; describe more complex techniques in the Methods section.*
☐	☒	A description of all covariates tested
☐	☒	A description of any assumptions or corrections, such as tests of normality and adjustment for multiple comparisons
☐	☒	A full description of the statistical parameters including central tendency (e.g. means) or other basic estimates (e.g. regression coefficient) AND variation (e.g. standard deviation) or associated estimates of uncertainty (e.g. confidence intervals)
☐	☒	For null hypothesis testing, the test statistic (e.g. F, t, r) with confidence intervals, effect sizes, degrees of freedom and P value noted<br>*Give P values as exact values whenever suitable.*
☒	☐	For Bayesian analysis, information on the choice of priors and Markov chain Monte Carlo settings
☒	☐	For hierarchical and complex designs, identification of the appropriate level for tests and full reporting of outcomes
☐	☒	Estimates of effect sizes (e.g. Cohen's *d*, Pearson's *r*), indicating how they were calculated

Our web collection on statistics for biologists contains articles on many of the points above.

Software and code

Policy information about availability of computer code

Data collection	ScanScope Console (version 8.2, LRI imaging AB, Vista CA, USA), Aperio image scope (version 8.0, Aperio, Vista Californien, USA), BiopixiQ (version 2.1.8, Gothenburg, Sweden), Luminex 100 IS diagnostic instrument (version 2.3, Austin, TX, USA), ImageLab software (version 6.1, Bio-Rad, USA). The single-cell transcriptome data was generated at the Eukaryotic Single-cell Genomics facility at Science for Life Laboratory in Stockholm, Sweden.
Data analysis	Central custom scripts are deposited at Zenodo https://doi.org/10.5281/zenodo.10420614. Additional used software are: SIMCA-P (version 14.1, Umetrics, Sweden), SPSS (version 22.0, IBM Corp., USA), GraphPad Prism (version 8, GraphPad Software, USA), BD FACSDiva (version 9.0.1, BD Biosciences, USA), STAR (version 2.5.1b52), Space Ranger software (version 1.0.0, 10X, USA) and R (version 4.2.2, Austria) packages of Seurat (version 3.2.3), ggforestplot (version 0.1.0), pheatmap (version 1.0.12), edgeR(version 3.40.2), sva (version 3.46.0) and ggplot2 (version 3.4.2).

For manuscripts utilizing custom algorithms or software that are central to the research but not yet described in published literature, software must be made available to editors and reviewers. We strongly encourage code deposition in a community repository (e.g. GitHub). See the Nature Portfolio guidelines for submitting code & software for further information.

Data

Policy information about availability of data

All manuscripts must include a data availability statement. This statement should provide the following information, where applicable:

- Accession codes, unique identifiers, or web links for publicly available datasets
- A description of any restrictions on data availability
- For clinical datasets or third party data, please ensure that the statement adheres to our policy

Individual data regarding living humans cannot be publicly available due to the sensitive nature of the data regulated by European privacy laws (GDPR). It migtht be accessed for the sole purpose of replicating the procedures and results presented in the article and providing that the data transfer is in agreement with European Union legislation on the general data protection regulation and decisions by the ethical review board of Sweden, the Region Skåne and the Lund University. The datasets are available with restricted access in the Swedish national infrastructure Science for Life Laboratory repository, in the DOI: https://doi.org/10.17044/scilifelab.26063056 . This link includes the terms of data accessibility by request to the corresponding author (Andreas.Edsfeldt@med.lu.se) in up to 6 weeks.

An independent single cell data set from human carotid plaques used in this study are available in the Gene Expression Omnibus database under accession code GSE159677 [https://www.ncbi.nlm.nih.gov/geo/query/acc.cgi?acc=GSE159677] and can be retrieved through logging in to PlaqView [https://www.plaqview.com/data].

The Cell Ranger hg38 reference genome refdata-cellranger-GRCh38-3.0.0 is available at http://cf.10xgenomics.com/supp/cell-exp/refdata-cellranger-GRCh38-3.0.0.tar.gz.

Source data are provided with this paper.

Research involving human participants, their data, or biological material

Policy information about studies with human participants or human data. See also policy information about sex, gender (identity/presentation), and sexual orientation and race, ethnicity and racism.

Reporting on sex and gender	The term sex (biological attribute), identified by the Swedish personal number, is used throughout the text. Sex was considered in the study design and reported results were, whenever possible, adjusted for sex. The current study includes both sexes, as the disease of interest affect both males and females.
Reporting on race, ethnicity, or other socially relevant groupings	No
Population characteristics	Patients, mean age 70 years, undergoing carotid endarterectomy due to advanced carotid atherosclerosis at the Vascular Department (Skåne University Hospital) were Included. Approximately 1/3 of the cohort was female. The ratio of type 2 diabetes and non-diabetes subjects was about 1:2. The detailed clinical characteristics are provided in the Supplementary Table 1.
Recruitment	All patients, with type 2 diabetes or without diabetes, undergoing carotid endarterectomy due to advanced carotid atherosclerosis at the Vascular Department at Skåne University Hospital were asked about their willingness to participate in the study. All patients who gave both oral and written informed consent were recruited. Individuals younger than 18 years of age and individuals not able to provide informed consent were excluded.<br>It is important to note that participants who volunteer for research studies might generally be more health-conscious compared to a broader population which could affect the general applicability of the study results.
Ethics oversight	Written informed consent was given by each patient. The study followed the declaration of Helsinki and was approved by the local ethical committee in Lund (472/2005; 2014/904; 2017/89; 2018/63)

Note that full information on the approval of the study protocol must also be provided in the manuscript.

Field-specific reporting

Please select the one below that is the best fit for your research. If you are not sure, read the appropriate sections before making your selection.

☒ Life sciences ☐ Behavioural & social sciences ☐ Ecological, evolutionary & environmental sciences

For a reference copy of the document with all sections, see nature.com/documents/nr-reporting-summary-flat.pdf

Life sciences study design

All studies must disclose on these points even when the disclosure is negative.

Sample size	A Monte Carlo simulation was conducted to estimate the required sample size for detecting a medium effect size (Cohen's d = 0.5) with a power of 0.8 when comparing two groups. The analysis revealed that a minimum of 70 samples per group is necessary to achieve the desired statistical power. Therefore, we recruited more than 70 individuals with T2D and over 140 individuals without diabetes. In total, 219 carotid plaques were included in the analyses.<br>For in vitro studies no statistical method was used to predetermined sample size. Sample size were based on our past experience with experimental models and treatment outcomes in the cell line used in current study.
Data exclusions	Outliers, significantly deviating data points, can emerge from measurement variability or errors, potentially skewing analysis and leading to

false conclusions. The ROUT method was employed to identify potential outliers, safeguarding the statistical findings' accuracy, reliability, and validity. The ROUT method operates on the principle of the False Discovery Rate (FDR), allowing for the specification of Q, which represents the maximum acceptable FDR. In this study, a Q value of 1% was employed, as recommended by Prism (GraphPad Software, Boston, USA) and supported by the literature (PMID: 16526949).

Replication

The findings were validated in an independent dataset using 10x Visium spatial sequencing on nine human carotid plaques from the Carotid Plaque Imaging Project biobank. The identifed cell types and TGFB isoform expressions were further confirmed using an independent and publicly available single cell RNA sequencing data of human carotid plaques (n=3 patient samples).
For in vitro studies, the findings were verified by performing experiments on at least two biological replicates, unless otherwise stated in the figure legends. All in vitro experiments were repeated at least three times.

Randomization

The study aimed to explore biological differences in the atherosclerotic disease comparing individuals without diabetes and those diagnosed with type 2 diabetes which were accepted for surgical removal of atherosclerotic carotid plaques. Given the specific aims of this study, randomization would not have been feasible.

Also randomization for different experimental groups for in-vitro studies was not applicable as they were carried out on commercially available cell lines.

Blinding

Plaque tissue analyses were fully blinded.
Blinding was not feasible for the in vitro studies as the individual investigators who preformed the experiments had to be aware of the experimental groups and treatments.
Blinding of investigator was also not relevant when performing the computational bioinformatic analyses of RNAseq data. However, all RNAseq analyses were data driven and unsupervised unless otherwise clearly stated in the methods section. The single cell RNAseq clustering analyses were performed without prior information about number of expected cell types .

Reporting for specific materials, systems and methods

We require information from authors about some types of materials, experimental systems and methods used in many studies. Here, indicate whether each material, system or method listed is relevant to your study. If you are not sure if a list item applies to your research, read the appropriate section before selecting a response.

Materials & experimental systems

n/a	Involved in the study	
☐	☒	Antibodies
☐	☒	Eukaryotic cell lines
☒	☐	Palaeontology and archaeology
☒	☐	Animals and other organisms
☐	☒	Clinical data
☒	☐	Dual use research of concern
☒	☐	Plants

Methods

n/a	Involved in the study	
☒	☐	ChIP-seq
☐	☒	Flow cytometry
☒	☐	MRI-based neuroimaging

Antibodies

Antibodies used

The following commercially available antibodies were used. Information about suppliers, catalogue number and clones are listed below whenever available. Antibody dilutions are provided in corresponding method sections of the main manuscript.
1. Anti-p-SMAD3 (phospho S423 + S425): Abcam, ab52903
2. Anti-p-SMAD2 (phospho S467): Abcam, ab53100
3. Anti-SMAD3: Abcam, ab40854
4. Anti-SMAD2: Abcam, ab40855
5. Anti-GAPDH: Abcam, ab8245
6. PE/Cyanine7 anti-human CD235a (Glycophorin A), clone HI264: Biolegend, 349111
7. APC anti-human CD45, clone HI30: Biolegend, 304012
8. Anti-human smooth muscle actin, Clone 1A4: Agilent (Formerly DakoCytomation), M0851
9. Anti-human CD68, Clone KP: Agilent (Formerly DakoCytomation), M0814
10. Anti-human CD235a, Glycophorin A, Clone JC159: Agilent (Formerly DakoCytomation), M0819

Validation

Validation statements about each antibody listed above can be found in the links provided here:

1. Validated by manufacturer for western blot application in human cell lines and used in 14 publications for WB on human samples based on their website. https://www.abcam.com/en-se/search?facets.target=SMAD3&facets.application=WB&facets.reactiveSpecies=Human&sorting=relevance&keywords=Anti-p-SMAD3+%28phospho+S423+%2B+S425%29+ab52903&productcode=AB52903&view=publications
2. Validated by manufacturer for western blot application in human cell lines and used in 3 publications for WB on human samples based on their website. https://www.abcam.com/en-se/search?facets.target=SMAD2&facets.application=WB&facets.reactiveSpecies=Human&sorting=relevance&keywords=Anti-p-SMAD2+%

28phospho+S467%29+ab53100&productcode=AB53100&view=publications
3. Validated by manufacturer for western blot application in human cell lines and used in 8 publications for WB on human samples based on their website. https://www.abcam.com/en-se/search?facets.target=SMAD3&facets.application=WB&facets.reactiveSpecies=Human&sorting=relevance&keywords=Anti-SMAD3+ab40854&productcode=AB40854&view=publications
4. Validated by manufacturer for western blot application in human cell lines and used in 3 publications for WB on human samples based on their website. https://www.abcam.com/en-se/search?facets.target=SMAD2&facets.application=WB&facets.reactiveSpecies=Human&sorting=relevance&keywords=Anti-SMAD2+ab40855&productcode=AB40855&view=publications
5. Validated by manufacturer for western blot application in human cell lines and used in 130 publications for WB on human samples based on their website. https://www.abcam.com/en-se/search?facets.target=GAPDH&facets.application=WB&facets.reactiveSpecies=Human&sorting=relevance&keywords=Anti-GAPDH+ab8245&productcode=AB8245&view=publications
6. Validated by manufacturer for FC application on human cells and used in 7 publications for similar application based on their website. https://www.biolegend.com/fr-lu/products/pe-cyanine7-anti-human-cd235a-glycophorin-a-antibody-9003
7. Validated by manufacturer for FC application on human cells and used in 73 publications for similar application based on their website. https://www.biolegend.com/fr-lu/products/apc-anti-human-cd45-antibody-705
8. Validated by Human protein atlas for IHC applications across 44 normal tissue samples using enhanced orthogonal validation method. Human Protein Atlas Number: CAB000002. https://www.proteinatlas.org/ENSG00000107796-ACTA2/summary/antibody
9. Validated by Human protein atlas for IHC applications across 44 normal tissue samples using enhanced orthogonal validation method. Human Protein Atlas Number: CAB000051. https://www.proteinatlas.org/ENSG00000129226-CD68/summary/antibody#IHC
10. Validated by manufacturer for IHC use in human samples https://www.agilent.com/cs/library/packageinsert/public/SSM0819CEEFG_02.pdf . Used by following publication: Erber WN, McLachlan J, Cordell JL, Turley H, Reid M, Mason DY. A new monoclonal antibody (JC159) that detects glycophorin A for the diagnosis of erythroleukaemia. Hematol Rev 1991;5:113-20.

Eukaryotic cell lines

Policy information about cell lines and Sex and Gender in Research

Cell line source(s)	Human Coronary Artery Smooth Muscle Cells (HCASMC; Cat#C-017-5C, ThermoFisher, New York, USA)
Authentication	HCASMC is a commercial cell line which was not authenticated by us but were used in several cited reports.
Mycoplasma contamination	Mycoplasma contamination was not detected.
Commonly misidentified lines (See ICLAC register)	No commonly misidentified cell lines were used in the current study

Clinical data

Policy information about clinical studies
All manuscripts should comply with the ICMJEguidelines for publication of clinical research and a completedCONSORT checklist must be included with all submissions.

Clinical trial registration	NCT05821894
Study protocol	Prospective, observational cohort study. Full trial protocol can be accessed via https://classic.clinicaltrials.gov/ProvidedDocs/94/NCT05821894/Prot_SAP_000.pdf
Data collection	Clinical data, follow-up data, atherosclerotic plaque tissue and blood was collected as detailed in the methods section. The study target population is patients with severe atherosclerotic carotid stenosis undergoing carotid endarterectomy at the Vascular Dept. of Skåne University Hospital (SUS), Malmö, Sweden. The inclusion started in 2005 and is still ongoing.
Outcomes	Postoperative myocardial infarction, stroke, transient ischaemic attack, amaurosis fugax and cardiovascular death according to Swedish National Registers. If patients suffered from more than one event, only the first chronological event was included in the analyses.

Plants

Seed stocks	N/A
Novel plant genotypes	N/A
Authentication	N/A

Flow Cytometry

Plots

Confirm that:

- [x] The axis labels state the marker and fluorochrome used (e.g. CD4-FITC).
- [x] The axis scales are clearly visible. Include numbers along axes only for bottom left plot of group (a 'group' is an analysis of identical markers).
- [x] All plots are contour plots with outliers or pseudocolor plots.
- [x] A numerical value for number of cells or percentage (with statistics) is provided.

Methodology

Sample preparation	The carotid plaque was taken immediately after surgical removal and placed in RPMI 1640 media. Subsequently, plaque tissue was cleaned of calcified areas, minced and placed into an enzymatic digestion cocktail consisting of collagenase type I (400 units/ml, Sigma C9722), elastase type III (5 units/ml, Worthington, LS006365), and DNase (300 units/ml, Sigma D5025), with 1 mg/ml soybean trypsin inhibitor (Sigma T6522), 2.5 µg/ml polymixin B (Sigma), and 2 mM CaCl2, in RPMI medium 1640 with 5% FBS). The suspension was incubated at 37 °C for 30 min with continuous agitation. After incubation cell suspension was pipetted up and down to break remaining tissue. Thereafter the cell suspension was strained using 100µm strainer and pelleted by centrifugation at 500g for 5min. The cells were then suspended in fresh RPMI 1640 media. For sorting purpose, the isolated cells were washed, stained with MitoTracker™ Green FM (Thermofisher scientific, Cat# M7514) (50nM 30 min at RT), FC blocked (Biolegend, Cat# 422302) (1:33 15min at RT), and subsequently incubated with antibody cocktail consisting of PE/Cy7 anti-human CD235a (Glycophorin A, Biolegend, Cat# 349111), and APC anti-human CD45 (Biolegend, Cat# 304012) antibodies at 1:100 dilution for 30 min at 4oC. Finally the cells were washed, resuspended in PBS containing viability dye 7-AAD (Biolegend, Cat# 420403) at 1:100 dilution and processed immediately for FACS sorting
Instrument	BD FACSAria III cell sorter (BD Biosciences)
Software	BD FACSDiva v9.0.1 (BD Biosciences)
Cell population abundance	Detailed in supl fig 2
Gating strategy	Provided in detail in Supplementary methods and supplementary figure 2. Live (7AAD, Biolegend, Cat# 420403)->singlets->CD45+ (anti-human CD45 (Biolegend, Cat# 304012) or CD45-. CD45- were further gated as CD235- (Glycophorin A, Biolegend, Cat# 349111) and Mtiotracker+ (Thermofisher scientific, Cat# M7514)

- [x] Tick this box to confirm that a figure exemplifying the gating strategy is provided in the Supplementary Information.
