# Supplementary material for: Author Correction: Dysregulation of MMP2-dependent TGF-ß2 activation impairs fibrous cap formation in type 2 diabetes-associated atherosclerosis
Source: Nat Commun. 2025 Feb 12;16:1567. doi: 10.1038/s41467-025-56969-6 (PMC11821807; doi:10.1038/s41467-025-56969-6)
Supplement: Supplementary file 1 — Updated Supplementary Information [file 41467_2025_56969_MOESM1_ESM.pdf]

SUPPLEMENTARY INFORMATION

Dysregulation of MMP2-dependent TGF-ß2 activation impairs fibrous cap formation in type 2 diabetes-associated atherosclerosis

Pratibha Singh[1#], Jiangming Sun[1#], Michele Cavalera[1], Dania Al-Sharify,[1] Frank Matthes[1], Mohammad Barghouth[1], Christoffer Tengryd[1], Pontus Dunér[2], Ana Persson[1], Lena Sundius[1], Mihaela Nitulescu[1], Eva Bengtsson[2,3,4], Sara Rattik[2], Daniel Engelbertsen[2], Marju Orho-Melander[2], Jan Nilsson[2], Claudia Monaco[5], Isabel Goncalves [1,6‡], Andreas Edsfeldt *[1,6,7‡]

[#] *These authors contributed equally: Pratibha Singh and Jiangming Sun*
[‡] *These authors jointly supervised this work: Isabel Goncalves and Andreas Edsfeldt*

[1] Cardiovascular Research – Translational Studies, Lund University, Malmö, Sweden
[2] Department of Clinical Sciences Malmö, Lund University, Malmö, Sweden
[3] Department of Biomedical Science, Malmö University, Malmö, Sweden
[4] Biofilms – Research Center for Biointerfaces, Malmö University, Malmö, Sweden
[5] Kennedy Institute of Rheumatology, Nuffield Department of Orthopaedics, Rheumatology and Musculoskeletal Sciences, University of Oxford, United Kingdom
[6] Department of Cardiology, University Hospital of Skåne, Lund/Malmö, Sweden
[7] Wallenberg Centre for molecular medicine, Lund University, Sweden

Supplementary Fig. 1.

a)

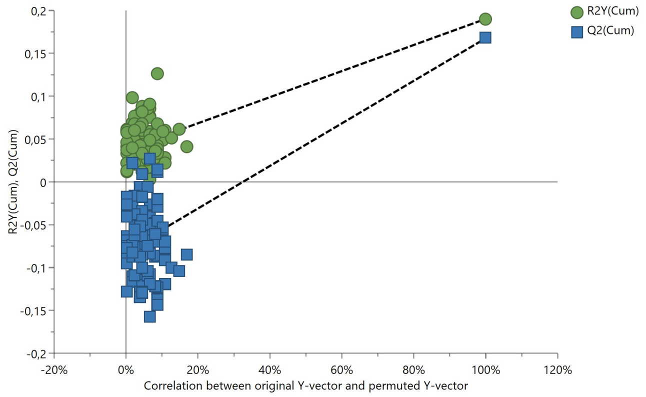

b)

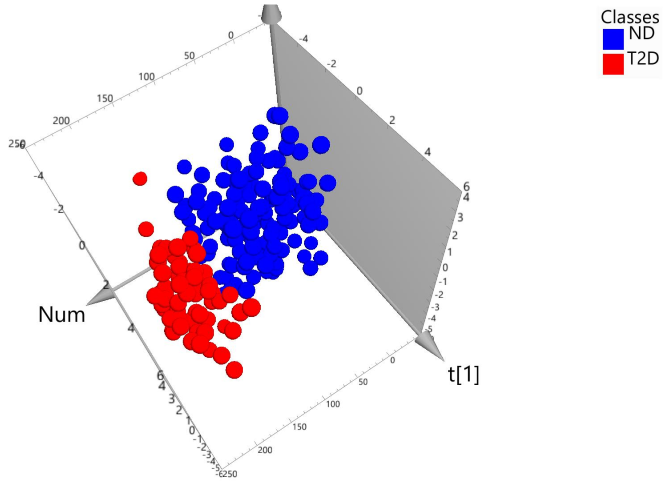

Supplementary Fig 1. a) Validation of the OPLS-DA model using permutation testing (n=100 tests). Each symbol represents a permutation result, R^2 is represented by green dots and Q^2 by blue squares. The 100-permutation test showed no overfitting in the OPLS-DA model (Q^2 = (0.0, -0.0783)). **b)** 3D score plot of the OPLS-DA data (15 growth factors in plaque tissue homogenates) of type 2 diabetes (T2D) and no diabetes (ND) plaques (n=218 patient samples; cross validation-ANOVA p-value=1.1e^{-6}). A clear separation of T2D plaques (red circles) from ND plaques (blue circles) was observed in the 3D view. ND, no diabetes; T2D, type 2 diabetes. Source data are provided in a source data file.

Supplementary Fig. 2.

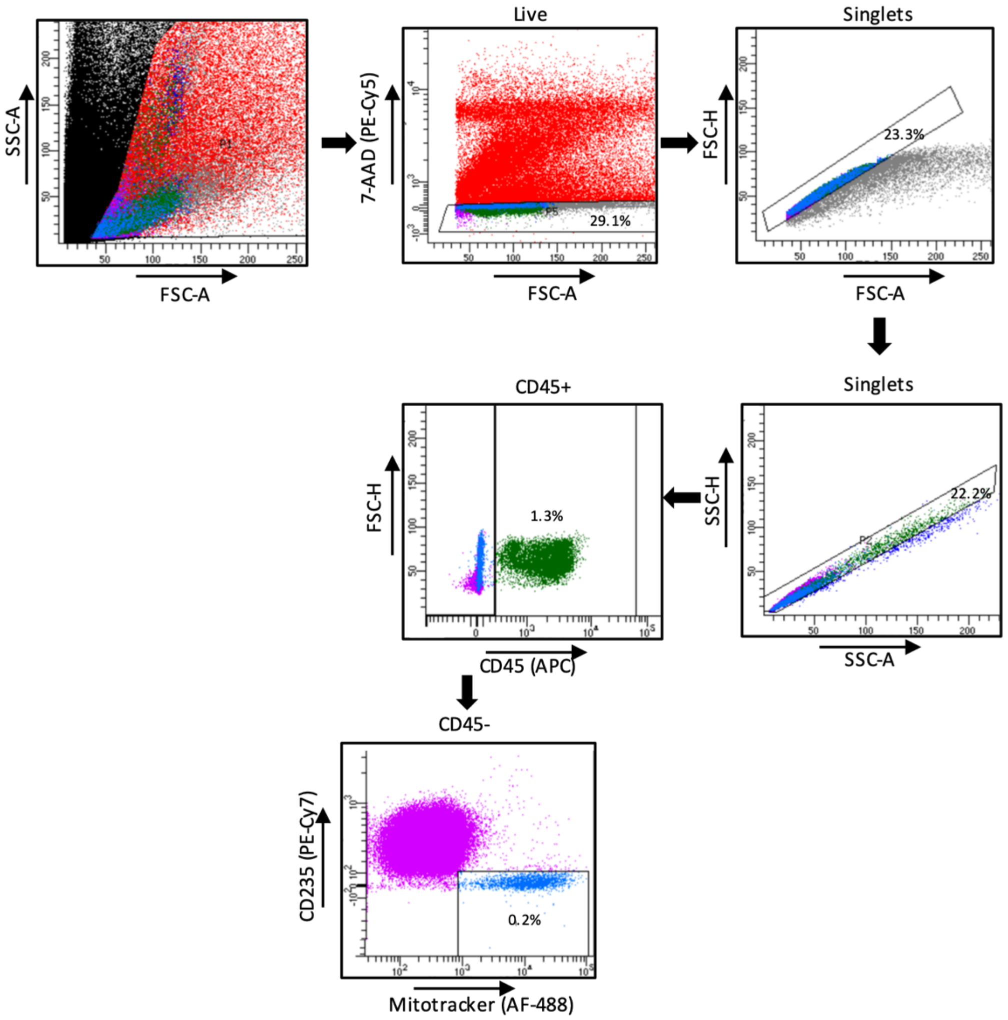

Supplementary Fig 2. Gating strategy used for sorting of $CD45^+$ and $CD45^-$ plaque cells for singel cell RNA sequencing. Apoptotic cells, red blood cells and doublets were excluded. Percentages refer to the total number of events.

Supplementary Fig. 3.

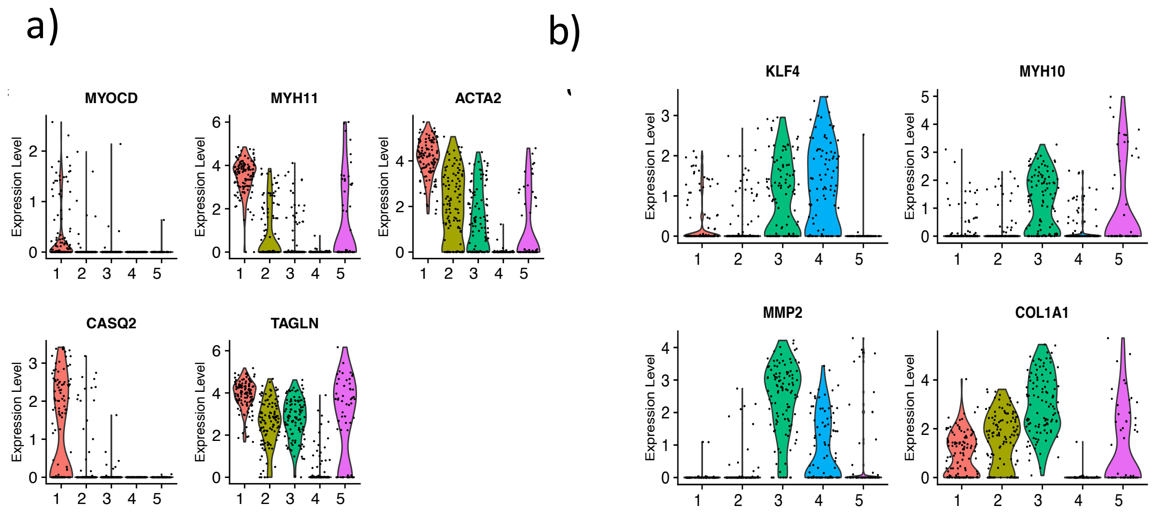

Supplementary Fig 3. Violin plots visualizing the expression of **a)** contractile and **b)** synthetic smooth muscle cell phenotype markers across $CD45^-$ cell clusters (n =489 cells). Clusters 1: contractile smooth muscle cells, Cluster 2: adipocyte like smooth muscle cells, Cluster 3: synthetic/fibroblast-like smooth muscle cells, Cluster 4: endothelial cells, Cluster 5: macrophage like smooth muscle cells. The gene expression is displayed in log-normalized counts. Source data are provided in a source data file.

Supplementary Fig. 4.

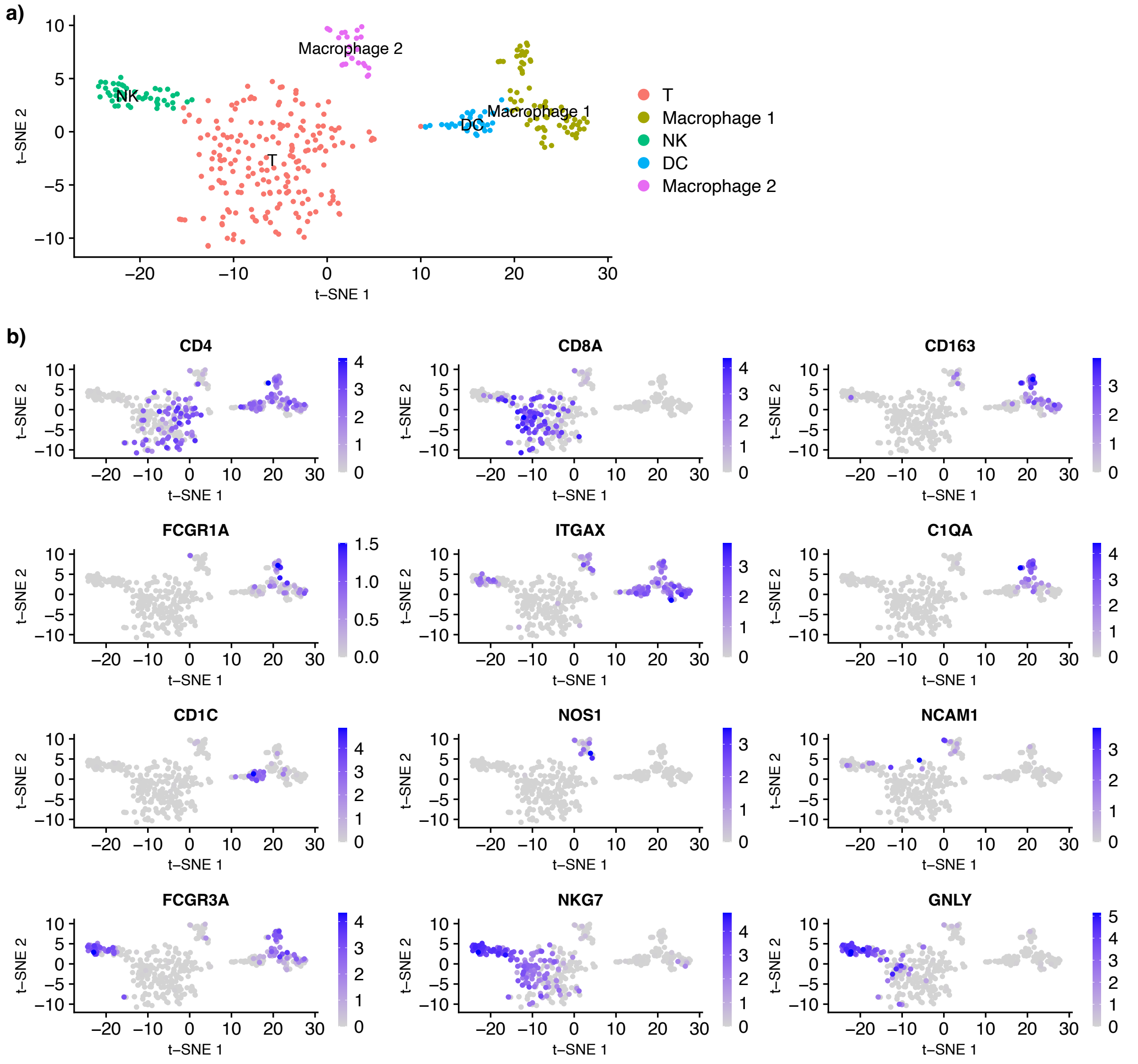

Supplementary Fig 4. Clustering analysis of CD45$^+$ carotid plaque cells (n =366 cells). **A)** Feature plot of CD45$^+$ cell clusters and **b)** expressions of respective cell marker genes. The colour scale represents log-normalized counts of gene expression.

Supplementary Fig. 5.

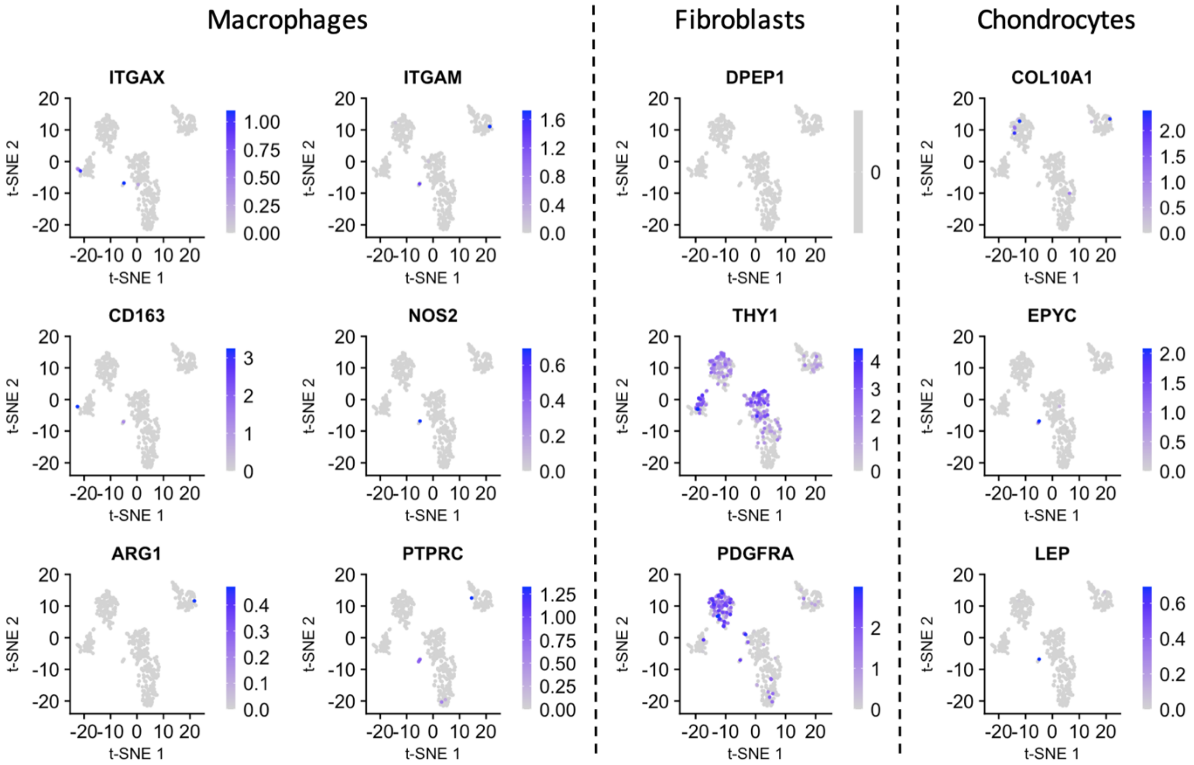

Supplementary Fig 5. t-SNE plots showing potential overlap of gene expression of prototypical markers for macrophages, fibroblasts and chondrocytes in the 5 identified CD45^{-} cell clusters (n =489 cells). The color scale represents log-normalized counts of gene expression.

Supplementary Fig. 6.

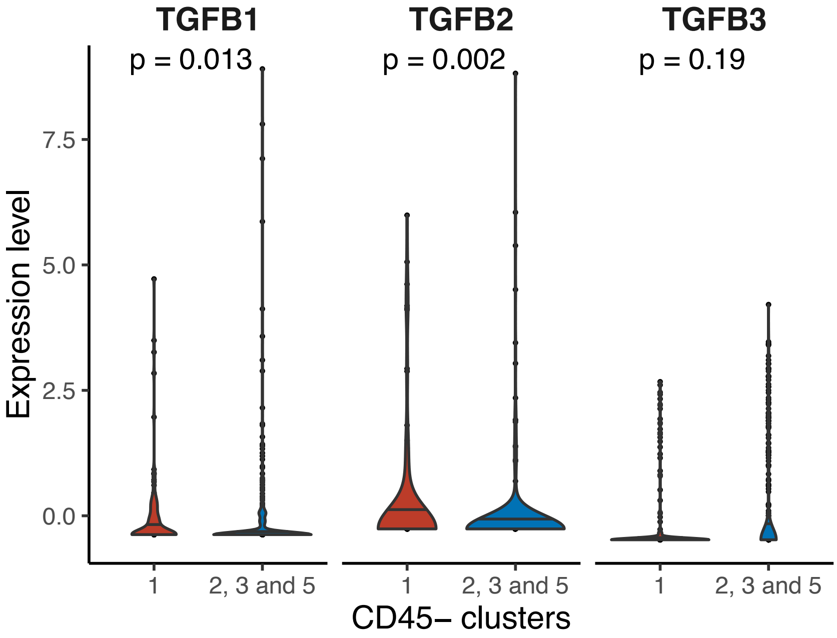

Supplementary Fig 6. Violin plot comparing expression of *TGFB* isoforms in the contractile vascular smooth muscle cells (VSMC; cluster 1) and other VSMC clusters (clusters 2, 3 and 5) (n =397 cells). *TGFB1* and *TGFB2* expression was significantly higher in contractile VSMC compared to the other three VSMC clusters. Two-sided Mann-Whitney U tests were used. VSMC: Vascular smooth muscle cells. Source data are provided in a source data file.

Supplementary Fig. 7.

a)

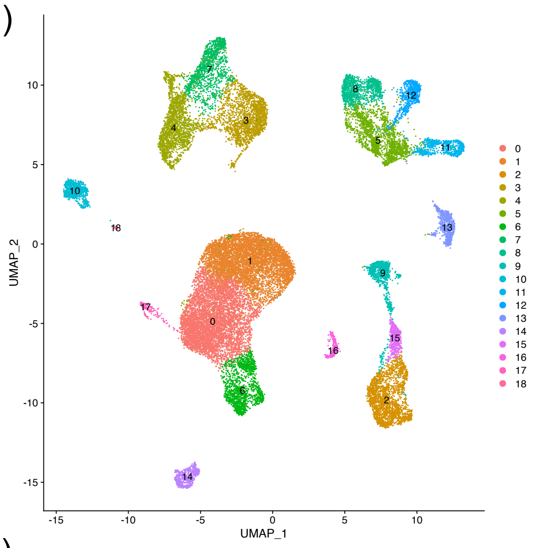

b)

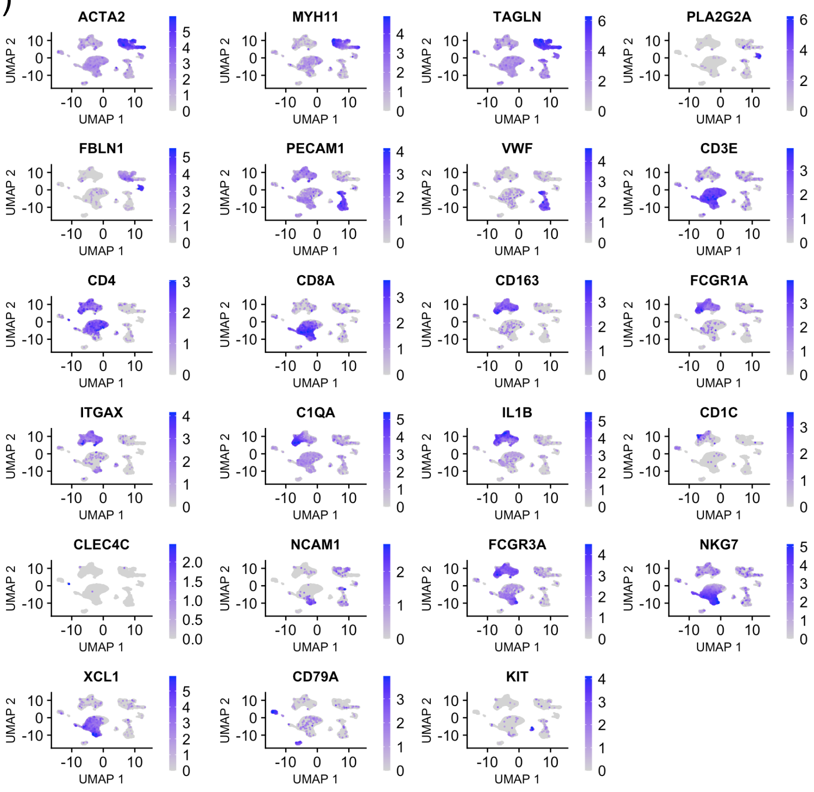

Supplementary Fig 7. UMAP visualization of all cells obtained from the PlaqView analysis of human carotid plaques scRNA-seq data (n=6 plaque regions)[1]. **a)** Identified clusters in PlaqView. **b)** Feature plot showing expression of main cell marker genes in respective clusters. The colour scale represents log-normalized counts of gene expression.

Supplementary Fig. 8.

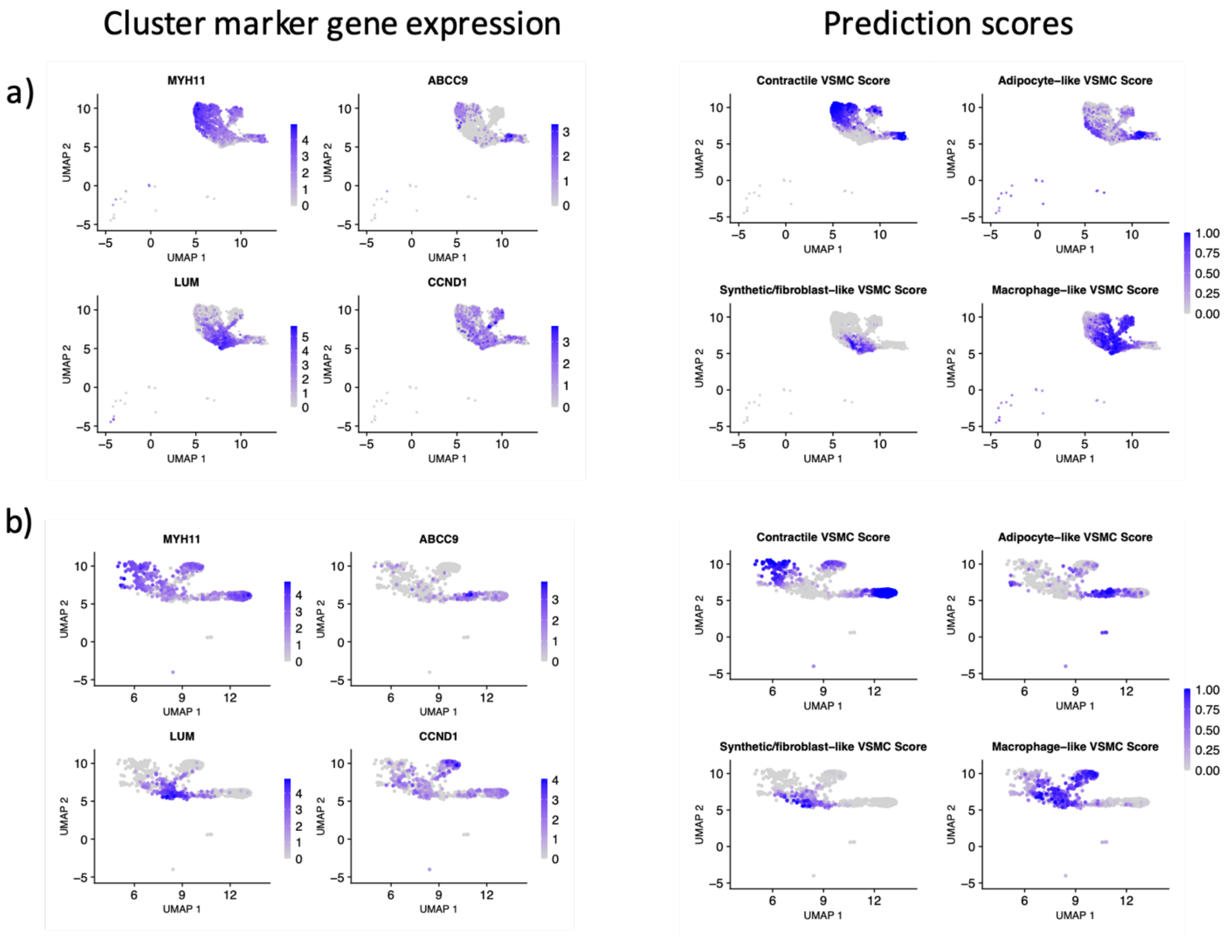

Supplementary Fig 8. Validation of vascular smooth muscle cell subtypes identified in the current study using an independent single cell RNA-sequencing dataset of human atherosclerotic plaque cells[1]. Feature plots showing expression (left) of main differentially expressed genes of vascular smooth muscle cell (VSMC) subtypes recognised in the current study, and their projection (right) on the VSMCs of **a)** the atherosclerotic core region (n=3 patient samples) and **b)** in the proximal adjacent region (n=3 patient samples). The colour scale on the left panel represents log-normalized counts of gene expression while the colour scale on the right panel represents the prediction scores.

Supplementary Fig. 9.

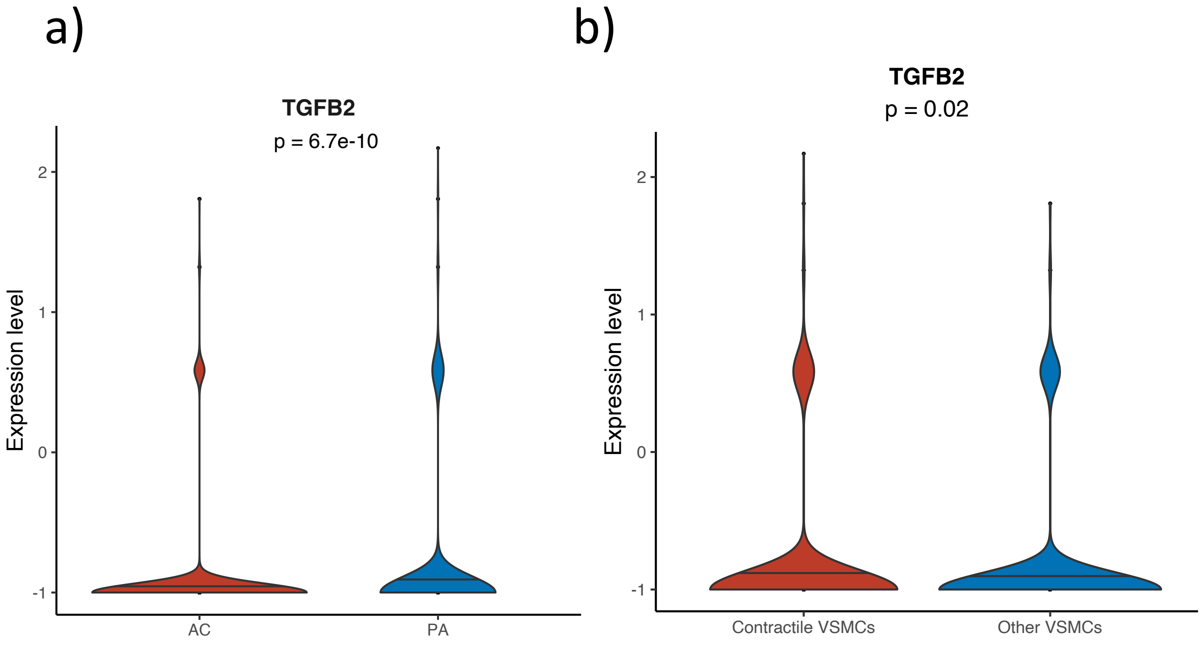

Supplementary Fig 9. a) Validation of *TGFB2* expression in a publicly available single cell RNA-sequencing dataset of human atherosclerotic plaques[1]. Violin plot showing higher vascular smooth muscle cell (VSMC) *TGFB2* expression detected in the proximal adjacent region (PA, blue) compared to the atherosclerotic core regions (AC, red) in this dataset (n=3 patient samples in each group). **b)** Violin plot showing higher *TGFB2* expression in the contractile VSMCs (red) compared to other VSMCs (blue) in the proximal adjacent region (n=3 patient samples). P-value is derived from a fixed-effect meta-analysis using the inverse-variance method to combine differences in *TGFB2* expression between contractile VSMCs and other VSMCs in proximal regions. Gene expression is the log2-transformed count with offset of 0.5. Gene expression of -1 denotes that 0 counts of such gene was detected. Two-sided Mann-Whitney U tests were used. Source data are provided in the source data file.

Supplementary Fig. 10.

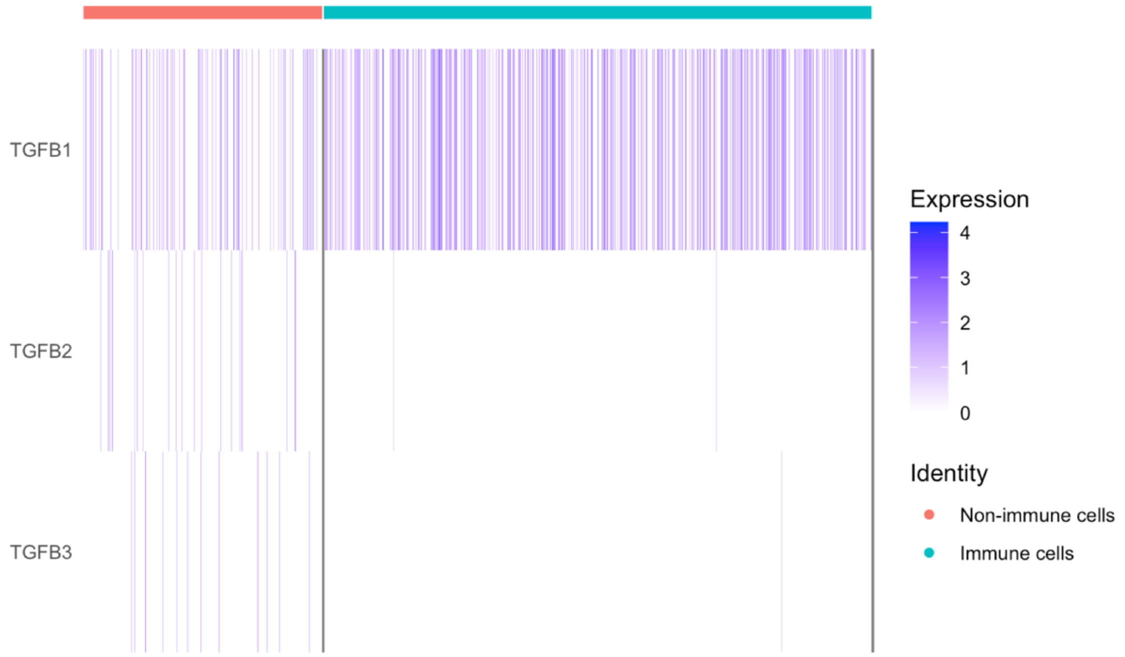

Supplementary Fig 10. Heatmap of the gene expression of *TGFB* isoforms in non-immune cells (salmon) and immune cells (cyan) (n=30 525 cells). Each column represents a cell where dark blue denotes a high expression level. The colour scale represents log-normalized counts of gene expression.

Supplementary Fig. 11.

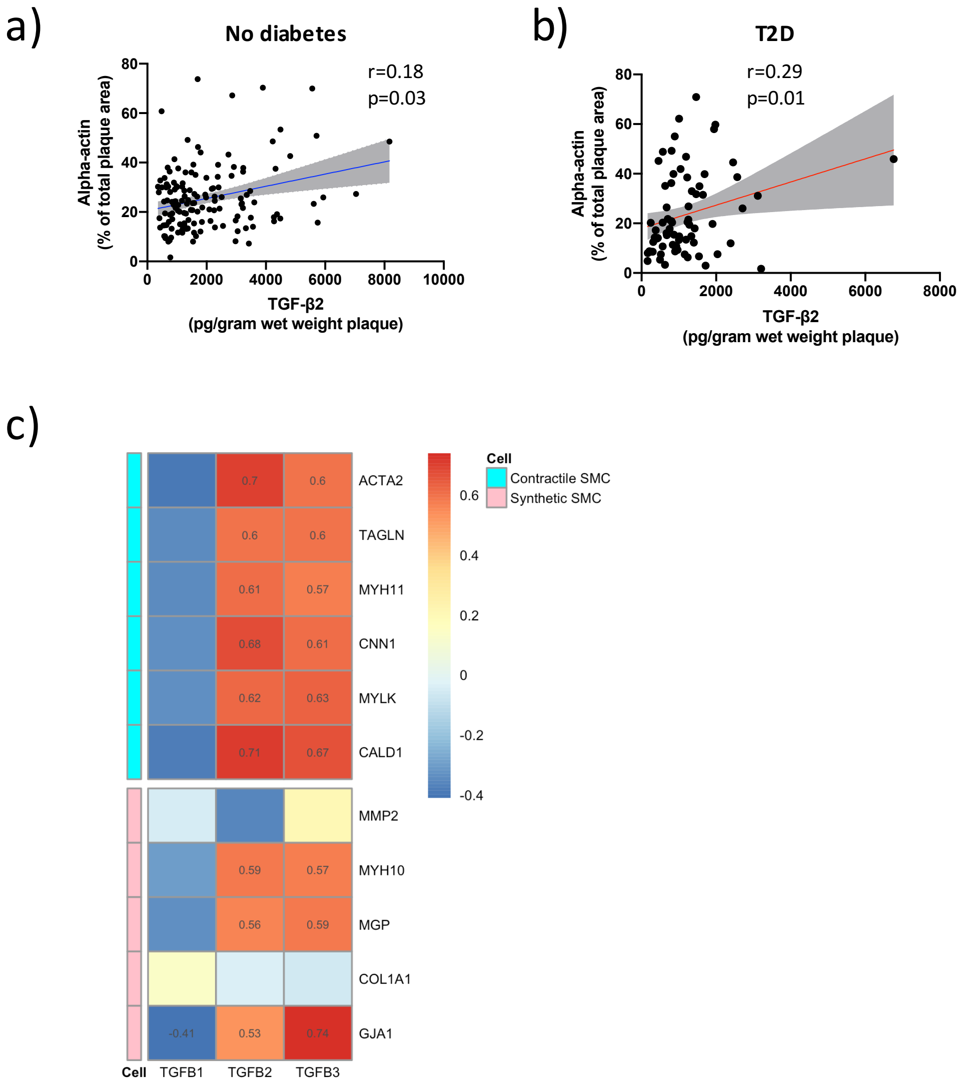

Supplementary Fig. 11. Plaque TGF-β2 protein and gene levels correlated to alpha-actin protein expression and *ACTA2* gene expression. Plaque levels of free TGF-β2 correlated with plaque area stained positive for smooth muscle alpha-actin in both **a)** plaques from patients without diabetes (n=146 patient samples) and **b)** plaques from patients with type 2 diabetes (T2D, n=72 patient samples). Values are visualized as scatter plots. The solid lines represents the linear regression line, and the grey bands show the 95% confidence interval. The Spearman correlation coefficients with two-sided p-values are presented. **c)** Heatmap visualizing correlations between gene expressions of *TGFB* isoforms and cell markers from bulk RNA-sequencing on human plaques. The Spearman correlation coefficients with two-sided p-values

is presented. Correlation coefficients (Spearman's Rho) are displayed if the p-value is less than 0.05. n=22 patient samples. The colour scale represents magnitude of correlation coefficient and ranged from negative (blue) to positive (red) correlations.

Supplementary Fig.12.

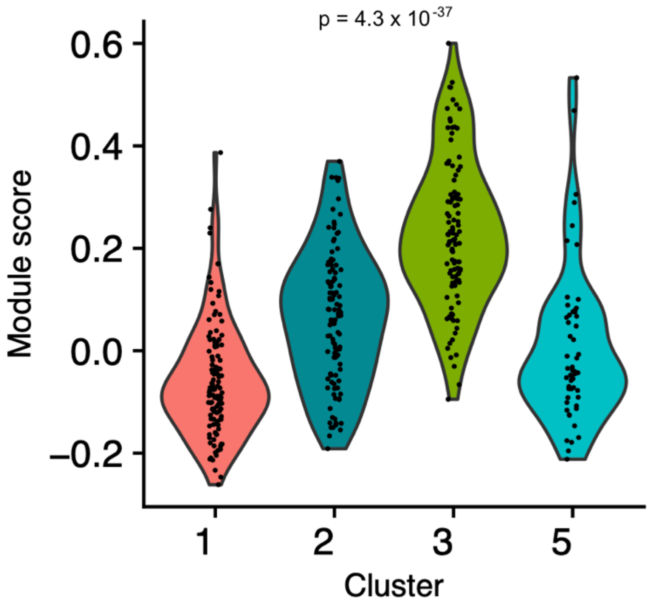

Supplementary Fig 12. Module scores of genes listed in the Collagen formation pathway (n cells=397). A higher module score is observed in the cluster 3 (synthetic/fibroblast-like vascular smooth muscle cells). Kruskal-Wallis test was used. Source data are provided in a source data file.

Supplementary Fig. 13.

a)

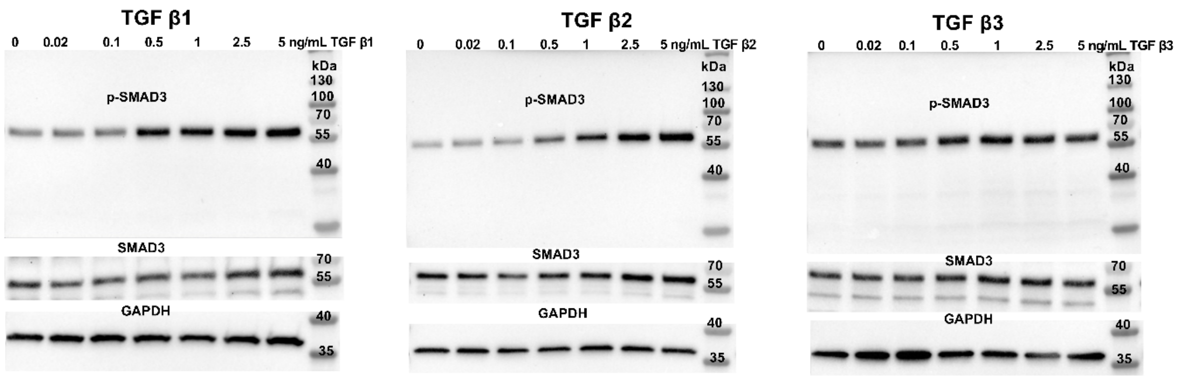

b)

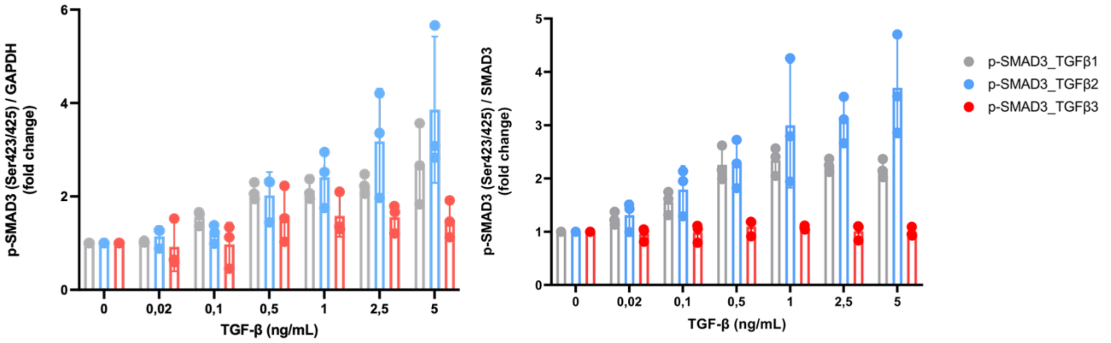

Supplementary Fig 13. TGF- β1, -β2 and -β3 induced Smad3 phosphorylation in synthetic human coronary arterial smooth muscle cells (HCASMC). **a)** HCASMC were treated for 15 min with up to 5 ng/ml of TGF-β1, - β2, or- β3 and examined for p-SMAD3 (Ser423/425), total SMAD and GAPDH proteins by western blot. **b)** TGF-β1(grey bars), - β2 (blue bars), or- β3 (red bars) proteins were normalized to total SMAD or GAPDH proteins. Presented values were normalized to their respective untreated controls. Results are expressed as mean ± standard deviation (SD). n=3 biological replicates from three independent experiments. Source data are provided in a source data file.

Supplementary Fig. 14.

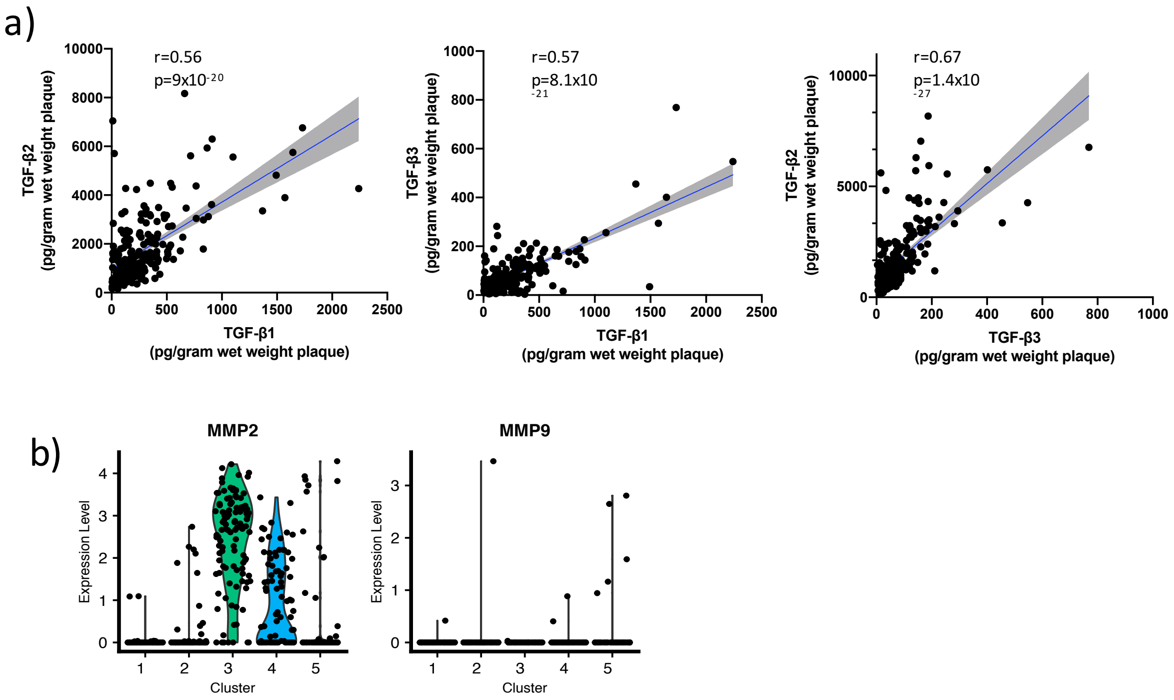

Supplementary Fig 14. a) All three TGF- β isoforms (free form) were positively correlated in plaque tissue homogenates. The data is presented as scatter plots where the blue lines represent the linear regression, and the grey bands show the 95% confidence intervals. The Spearman correlation coefficients with two-sided p-values are presented on the top. n=219 patient samples. **b)** Human plaque single cell RNA-sequencing identified that *MMP2* was mainly expressed by synthetic smooth muscle cells (cluster 3) and endothelial cells (cluster 4) whereas *MMP9* expression was only detected in a few CD45$^-$ cells. The gene expression is displayed as log-normalized counts in violin plots. TGF, transforming growth factor. MMP, matrix metalloproteinase.

Supplementary Fig. 15.

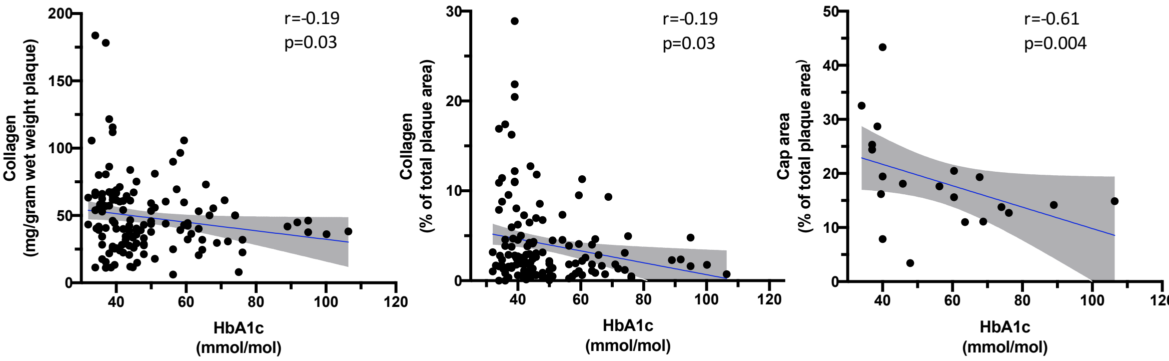

Supplementary Fig 15. Total plaque collagen (mg/g plaque weight, n=134 patient samples), collagen plaque area (% of total plaque area, n=132 patient samples) and fibrous cap area were all negatively correlated to plasma HbA1c levels (mmol/mol, n=18 patient samples). The data is visualized as scatter plots. Blue lines represent the linear regression line, and the grey bands show the 95% confidence intervals. Spearman correlation coefficients with two-sided p-values are shown. HbA1c, hemoglobin A1c.

Supplementary Fig 16.

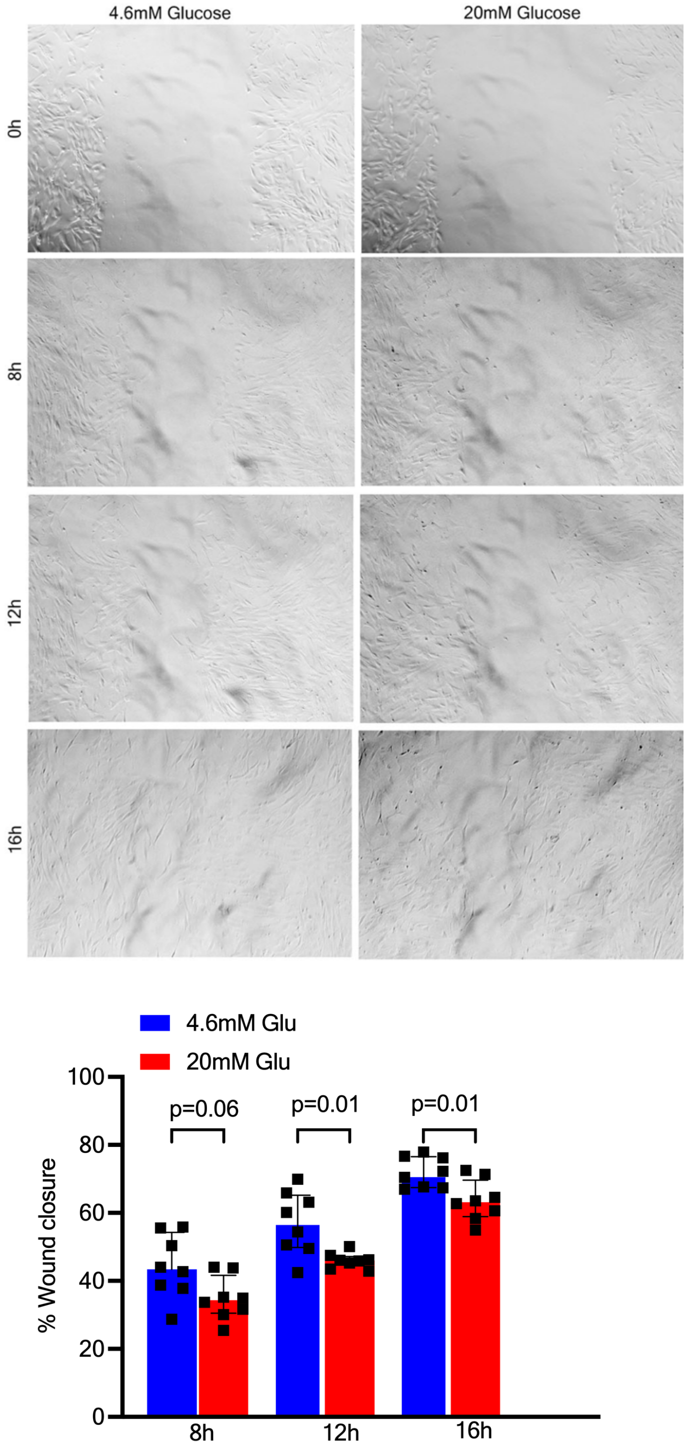

Supplementary Fig 16. Hyperglycemia reduced vascular smooth muscle cells migration. A wound scratch assay showed that hyperglycemia significantly reduced human coronary arterial smooth muscle cell (HCASMC) migration at 12 and 16 hours. Data is presented as median and

interquartile range, n=8 biological samples from 4 independent experiments. Two-sided Mann-Whitney U tests were used. Source data are provided in a source data file.

Supplementary Fig. 17

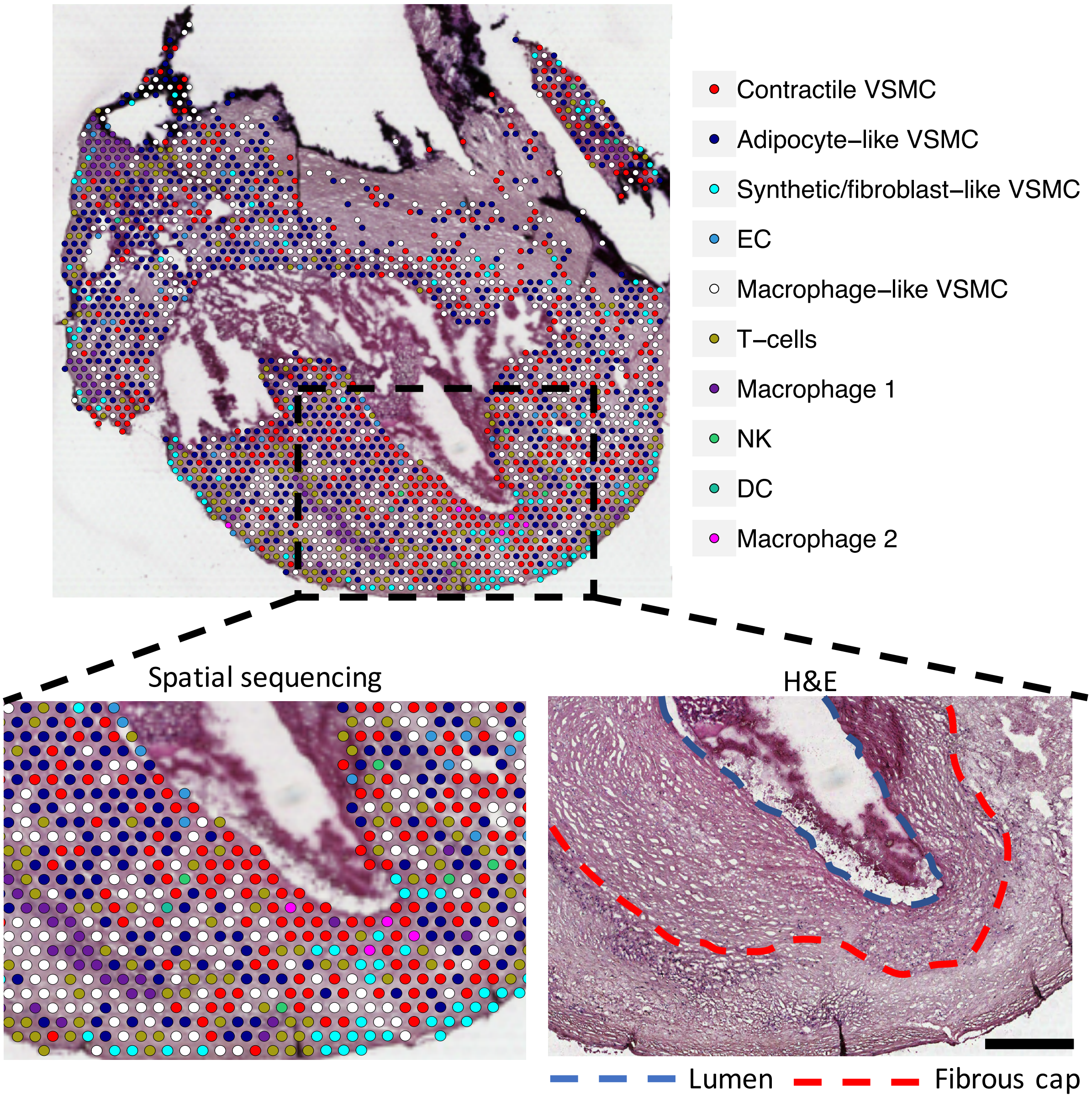

Supplementary Fig. 17. The top predicted cell type per spot (based on the single cell RNA sequencing data) showing the morphological distribution of both *PTPRC*$^{+}$ and *PTPRC*$^{-}$ cell phenotypes in a human carotid plaque. VSMC: vascular smooth muscle cells; EC: Endothelial cells; NK: Natural killer cells; DC: Dendritic cells, H&E: Haematoxylin and eosin. Scale bar 500µm. Blue dotted line marks the lumen. Red dotted line marks the fibrous cap.

Supplementary Fig. 18.

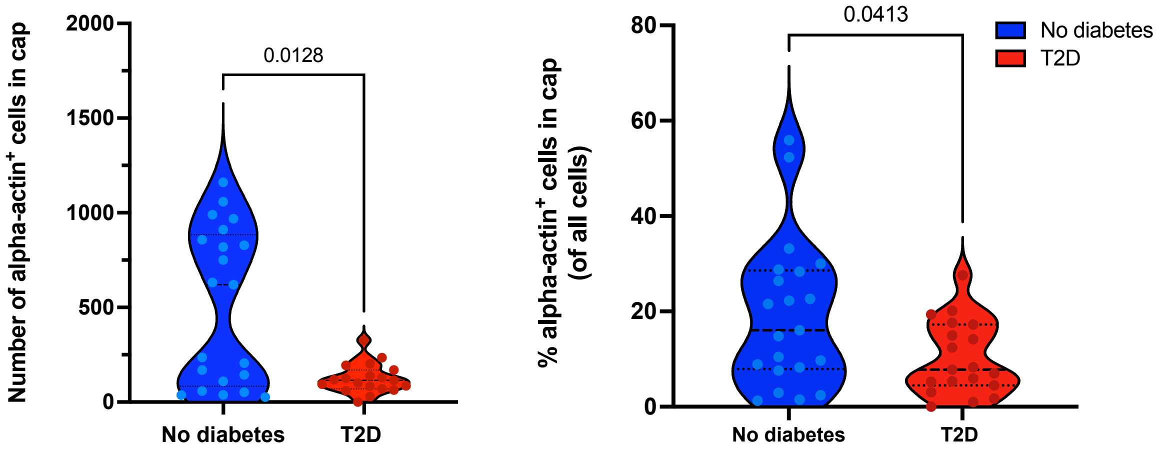

Supplementary Fig 18. Total number and % of alpha actin^{+} cells (of total cell count) in the fibrous caps were significantly lower in plaques from patient with type 2 diabetes (T2D) as assessed by immunohistochemistry. n=21 for the no diabetes group and n=19 for the T2D group. Two extreme outliers, identified by ROUTs test (Q=1%), were removed. Two-sided Mann-Whitney U tests were used. Data presented as violin plots with lines indicating median and interquartile range.

Supplementary Table 1. Top 15 marker genes for the cluster 1

Gene	Log2FC	p-value	Bonferroni-adjusted p-value
RERGL	3.12	4.4E-49	8.9E-45
ACTA2	2.93	2.8E-49	5.6E-45
MYH11	2.38	8.3E-54	1.7E-49
CASQ2	2.36	4.3E-36	8.7E-32
PLN	2.32	6.9E-46	1.4E-41
MCAM	2.11	2.6E-39	5.2E-35
NET1	2.09	8.3E-39	1.7E-34
LBH	1.95	1.4E-32	2.9E-28
C10orf10	1.87	9.9E-17	2.0E-12
HSPB8	1.79	5.3E-25	1.1E-20
RCAN2	1.73	2.5E-30	5.1E-26
TPM2	1.72	2.0E-38	4.0E-34
OR51E1	1.72	5.3E-32	1.1E-27
DSTN	1.70	1.0E-41	2.0E-37
CSRP1	1.68	5.1E-28	1.0E-23

Log2FC: log2-transformed fold change of gene expression comparing cluster 1 to all the other clusters (n =489 cells) of $CD45^-$ cells. Two-sided Wilcoxon Rank-Sum test was used.

Supplementary Table 2. Top 15 marker genes for the cluster 2

Gene	Log2FC	p-value	Bonferroni-adjusted p-value
CD36	2.67	9.2E-26	1.9E-21
RGS5	2.50	4.8E-40	9.7E-36
KCNJ8	2.13	1.0E-28	2.0E-24
ABCC9	2.01	7.8E-44	1.6E-39
PLXDC1	2.00	2.3E-29	4.7E-25
MYO1B	1.83	3.5E-26	7.1E-22
STEAP4	1.79	7.0E-27	1.4E-22
PDGFRB	1.74	1.7E-31	3.5E-27
TGFBI	1.67	7.1E-20	1.4E-15
ANO1	1.47	2.8E-30	5.7E-26
THY1	1.43	1.6E-15	3.3E-11
CCDC102B	1.43	4.0E-27	8.2E-23
LPL	1.43	7.7E-20	1.6E-15
TPPP3	1.41	1.6E-19	3.3E-15
PHLDA1	1.40	3.8E-20	7.7E-16

Log2FC: log2-transformed fold change of gene expression comparing cluster 2 to all the other clusters (n =489 cells) of $CD45^-$ cells. Two-sided Wilcoxon Rank-Sum test was used.

Supplementary Table 3. Top 15 marker genes for the cluster 3

Gene	Log2FC	p-value	Bonferroni -adjusted p-value
SFRP4	3.99	2.6E-61	5.3E-57
VCAN	3.68	7.0E-61	1.4E-56
LTBP2	3.54	3.5E-67	7.1E-63
LUM	3.47	3.3E-57	6.6E-53
FBLN1	3.46	1.8E-53	3.6E-49
SFRP2	3.36	3.3E-39	6.7E-35
THBS2	3.07	2.4E-53	4.9E-49
COL3A1	3.04	6.8E-31	1.4E-26
DCN	2.92	2.7E-39	5.4E-35
CCL19	2.87	6.8E-14	1.4E-09
MMP2	2.70	3.0E-48	6.2E-44
COL1A2	2.70	1.4E-40	2.8E-36
COL1A1	2.61	2.4E-34	4.8E-30
C7	2.48	1.8E-21	3.6E-17
CFH	2.48	9.1E-42	1.9E-37

Log2FC: log2-transformed fold change of gene expression comparing cluster 3 to all the other clusters (n =489 cells) of $CD45^-$ cells. Two-sided Wilcoxon Rank-Sum test was used.

Supplementary Table 4. Top 15 marker genes for the cluster 4

Gene	Log2FC	p-value	Bonferroni -adjusted p-value
VWF	5.33	3.4E-61	6.8E-57
PECAM1	4.57	3.1E-78	6.2E-74
CD74	4.53	5.0E-59	1.0E-54
ACKR1	4.31	2.8E-63	5.6E-59
PLVAP	4.17	8.7E-84	1.8E-79
SLCO2A1	4.03	5.9E-73	1.2E-68
RNASE1	3.69	8.2E-87	1.7E-82
HLA-DRB1	3.63	1.4E-80	2.8E-76
SELE	3.60	3.5E-46	7.2E-42
CD34	3.49	4.7E-68	9.6E-64
TSPAN7	3.38	1.9E-69	3.8E-65
PRCP	3.34	1.3E-41	2.7E-37
CLEC14A	3.34	1.1E-94	2.2E-90
HYAL2	3.32	4.4E-64	9.0E-60
RAMP2	3.15	2.6E-96	5.3E-92

Log2FC: log2-transformed fold change of gene expression comparing cluster 4 to all the other clusters (n =489 cells) of $CD45^-$ cells. Two-sided Wilcoxon Rank-Sum test was used.

Supplementary Table 5. Top 15 marker genes for the cluster 5

Gene	Log2FC	p-value	Bonferroni -adjusted p-value
FTL	3.95	7.5E-20	1.5E-15
SH3BGRL3	3.44	4.3E-13	8.8E-09
FTH1	2.93	3.4E-17	7.0E-13
ITGBL1	2.93	1.3E-06	2.6E-02
ANTXR1	2.81	2.8E-07	5.8E-03
TMSB4X	2.35	3.4E-27	6.9E-23
S100A10	2.34	2.2E-12	4.5E-08
UCHL1	2.17	1.4E-07	2.9E-03
S100A6	2.00	6.3E-22	1.3E-17
LGALS1	1.97	1.2E-16	2.4E-12
LYZ	1.83	5.0E-12	1.0E-07
CCL5	1.81	7.1E-08	1.5E-03
RPLP1	1.76	8.2E-22	1.7E-17
RGS1	1.66	1.1E-06	2.3E-02
S100A4	1.55	1.6E-07	3.3E-03

Log2FC: log2-transformed fold change of gene expression in the cluster 5 comparing to all the other clusters (n =489 cells) of $CD45^{-}$ cells. Two-sided Wilcoxon Rank-Sum test was used.

Supplementary Table 6. Multiple regression analysis showing associations between free TGF-β2 (dependent variable) plaque levels and clinical risk factors (n=219 patient samples).

Variable	Unstandardised coefficients		Standardised coefficients	t	P
	B	SE	β		
HbA1c (mmol/mol)	-26.6	9.5	-0.26	-2.8	0.006
HsCRP	-26.4	18.8	-0.13	-1.4	0.163
eGFR	3.0	67.2	0.06	0.4	0.678
BMI	-13.8	47.5	-0.04	-0.3	0.772
Total cholesterol (mmol/L)	-101.5	790.7	-0.08	-0.1	0.898
LDL (mmol/L)	160.3	792.8	0.11	0.2	0.840
Triglycerides (mmol/L)	-49.9	376..8	-0.03	-0.1	0.895
HDL (mmol/L)	-97.5	840.7	-0.02	-0.1	0.908
Age(years)	-24.3	19.4	-0.14	-1.2	0.214
Smoking	121.2	184.9	0.06	0.7	0.513
Hypertension	-486.5	330.6	-0.14	-1.5	0.144

HbA1c, hemoglobin A1c; HsCRP, high sensitive C-reactive protein; eGFR, estimated glomerular filtration rate; BMI, body mass index; LDL, low density lipoproteins; HDL, high density lipoproteins. Two-sided Student's t-test was used.

Supplementary Table 7. Clinical characteristics of the patients for spatial transcriptome analysis (n=9 patient samples) in human carotid atherosclerotic plaques.

	All	No T2D (n=5)	T2D (n=4)
Age(years, IQR)	68 (66-72)	68 (66-75)	67 (62-72)
Sex– Males (%)	9 (100)	5 (100%)	4 (100%)
Current Smoker (%)	3 (33.3)	2 (40)	1 (25)
BMI (IQR)	31.0 (25.6-33.0)	25.6 (24.4-33.0)	32.0 (30.2-33.2)
Degree of stenosis (%, IQR)	95 (90-95)	95 (90-95)	92.5 (87.5-95)
Hypertension (%)	9 (100)	5 (100)	4 (100)
hsCRP (mg/L, IQR)	2 (1.2-9.0)	9.0 (2-11)	1.7 (1.3-2.5)
HbA1c (mmol/mol, IQR)	41 (39-52)	39 (38-40)	53 (48-59)
Total cholesterol (IQR)	3.2 (3.0-3.7)	3.2 (3.1-3.9)	3.3 (3.0-3.6)
LDL (IQR)	2.0 (1.6-2.2)	2.0 (1.9-2.2)	1.9 (1.4-2.2)
HDL (IQR)	1.1 (1.0-1.3)	1.3 (1.0-1.4)	1.1 (1.0-1.1)
Triglycerides (IQR)	1.1 (1.0-1.7)	1.1 (1.0-1.4)	1.4 (1.0-1.9)
Blood pressure lowering treatment, n(%)	9 (100)	5 (46)	4 (100)
RAS inhibitor*, n(%)*	8 (89)	4 (80)	4 (100)
Beta blocker*, n(%)*	6 (67)	2 (40)	4 (100)
Statin treatment, n(%)	9 (100)	5 (100)	4 (100)

Values for continuous variables are presented as medians with interquartile ranges (IQR), while categorical variables are summarized as numbers and percentages.
HsCRP, high sensitive C-reactive protein; eGFR, estimated glomerular filtration rate; BMI, body mass index; HDL: high-density lipoprotein; LDL, low density lipoproteins; RAS, renin-angiotensin system. Plasma lipoproteins are presented as mmol/L.

Supplementary Table 8. Clinical characteristics of the study cohort.

	All	No T2D (n=147)	T2D (n=72)
Age(years)	70 (SD 8.7)	70 (SD 8.8)	70 (SD 8.4)
Sex– Males (%)	150 (68)	102 (69)	48 (66)
Smoking- current/ (%)	66 (30)	46 (31)	20 (28)
BMI	27 (SD 3.8)	26 (SD 3.6)	28 (SD 3.9)
Degree of stenosis(%)	90 (IQR 80-95)	90 (IQR 80-95)	90 (IQR 75-95)
Hypertension(%)	162 (74)	109 (74)	53 (74)
hsCRP (mg/L)	3.7 (IQR 1.9-6.3)	3.4 (IQR 1.9-6.3)	4.0 (IQR 1.9-6.3)
HbA1c (mmol/mol)	43 (IQR 38-55)	39 (IQR 36-41.7)	56 (46-65)
Plasma Lipoproteins(mmol/L)			
Total cholesterol	4.4 (SD 1.1)	4.5 (SD 1.1)	4.1 (SD 1.0)
LDL	2.5 (IQR 1.9-3.2)	2.6 (IQR 2.0-3.3)	2.2 (IQR 1.6-2.8)
HDL	1.1 (IQR 0.9-1.3)	1.1 (IQR 0.9-1.4)	1.0 (IQR 0.9-1.3)
Triglycerides	1.3 (IQR 1.0-1.8)	1.2 (IQR 0.9-1.8)	1.5 (IQR 1.0-2.0)
Blood glucose lowering treatment, n(%)			
Life style changes only	8 (4%)	-	8 (11%)
Oral glucose lowering treatment	40 (18%)	-	40 (56%)
Insulin	12 (5%)	-	12 (17%)
Insulin and oral glucose lowering	10 (5%)	-	10 (14%)
Blood pressure lowering treatment, n(%)			
RAS inhibitor	109 (50%)	67 (46%)	42 (58%)
Beta blocker	112 (51%)	71 (48%)	41 (57%)
Statin treatment, n(%)	187 (85%)	123 (84%)	64 (89%)

Values for continuous variables are reported as either medians with interquartile ranges (IQR) or means with standard deviations (SD), depending on the distribution of the data.
Categorical variables are expressed as numbers and percentages.
Information regarding current blood glucose treatment was missing for two patients.
HbA1c, hemoglobin A1c; HsCRP, high sensitive C-reactive protein; eGFR, estimated glomerular filtration rate; BMI, body mass index; LDL, low density lipoproteins; RAS, renin-angiotensin system.

Supplementary Table 9. TaqMan gene expression assays used in study.

TaqMan® Gene Expression Assays	Suppliers	Catalogue number
TGFB1	Thermo Fisher Scientific	Hs00998133_m1
TGFB2	Thermo Fisher Scientific	Hs00234244_m1
TGFB3	Thermo Fisher Scientific	Hs01086000_m1
ACTA2	Thermo Fisher Scientific	Hs00909449_m1
MYH11	Thermo Fisher Scientific	Hs00975796_m1
TAGLN	Thermo Fisher Scientific	Hs00162558_m1
CALD1	Thermo Fisher Scientific	Hs00189021_m1
COL1A1	Thermo Fisher Scientific	Hs00164004_m1
COL3A1	Thermo Fisher Scientific	Hs00943809_m1
COL4A1	Thermo Fisher Scientific	Hs00266237_m1

Supplementary references

1. Alsaigh, T, Evans, D, Frankel, D & Torkamani, A. Decoding the transcriptome of calcified atherosclerotic plaque at single-cell resolution. *Commun Biol* **5**, 1084 (2022).
